# Arbuscular Mycorrhizal Fungi Alleviate Drought Stress in Trifoliate Orange by Regulating H^+^-ATPase Activity and Gene Expression

**DOI:** 10.3389/fpls.2021.659694

**Published:** 2021-03-25

**Authors:** Hui-Qian Cheng, Ying-Ning Zou, Qiang-Sheng Wu, Kamil Kuča

**Affiliations:** ^1^College of Horticulture and Gardening, Yangtze University, Jingzhou, China; ^2^Department of Chemistry, Faculty of Science, University of Hradec Kralove, Hradec Kralove, Czechia

**Keywords:** citrus, H^+^-ATPase, mycorrhiza, proton pump, water deficit

## Abstract

A feature of arbuscular mycorrhiza is enhanced drought tolerance of host plants, although it is unclear whether host H^+^-ATPase activity and gene expression are involved in the physiological process. The present study aimed to investigate the effects of an arbuscular mycorrhizal fungus (AMF), *Funneliformis mosseae*, on H^+^-ATPase activity, and gene expression of trifoliate orange (*Poncirus trifoliata*) seedlings subjected to well-watered (WW) and drought stress (DS), together with the changes in leaf gas exchange, root morphology, soil pH value, and ammonium content. Soil drought treatment dramatically increased H^+^-ATPase activity of leaf and root, and AMF inoculation further strengthened the increased effect. A plasma membrane (PM) H^+^-ATPase gene of trifoliate orange, PtAHA2 (MW239123), was cloned. The *PtAHA2* expression was induced by mycorrhization in leaves and roots and also up-regulated by drought treatment in leaves of AMF-inoculated seedlings and in roots of AMF- and non-AMF-inoculated seedlings. And, the induced expression of *PtAHA2* under mycorrhization was more prominent under DS than under WW. Mycorrhizal plants also showed greater photosynthetic rate, stomatal conductance, intercellular CO_2_ concentration, and transpiration rate and better root volume and diameter than non-mycorrhizal plants under DS. AMF inoculation significantly increased leaf and root ammonium content, especially under DS, whereas it dramatically reduced soil pH value. In addition, H^+^-ATPase activity was significantly positively correlated with ammonium contents in leaves and roots, and root H^+^-ATPase activity was significantly negatively correlated with soil pH value. Our results concluded that AMF stimulated H^+^-ATPase activity and *PtAHA2* gene expression in response to DS, which resulted in great nutrient (e.g., ammonium) uptake and root growth, as well as low soil pH microenvironment.

## Introduction

Drought is one of the most serious environmental stresses, which severely restrains the growth and productivity of crop by destroying various physiological and biochemical processes such as nutrient absorption, photosynthesis, and cell metabolism (Zhang et al., 2020). As the initial site of damage after stress, changes in cell membrane structure negatively affect transport of various inorganic ions (Yoshida, 1991). Studies have demonstrated that cells regulated ion balances by changing the transmembrane transport of both ions and small molecules to maintain cell osmotic pressure under drought stress (DS) (Mak et al., 2014). Therefore, it is of profound significance to evaluate the transmembrane transport of key ions for revealing the mechanism of plants in response to DS.

Plasma membrane (PM) H^+^-ATPase is a kind of main membrane proteins widely existing in plant organelles (Małgorzata et al., 2018). Based on an electrical gradient inside and outside, H^+^ enters and exits the cell PM providing a driving force for the transport of small molecules, which is associated with mineral nutrient (NH_4_^+^) absorption, metabolite discharge, cytoplasmic pH regulation, cell growth, and stomata opening (Gaxiola et al., 2007). According to the “acid” growth theory, plant growth requires acidification of the cell wall space, and such acidification is derived by H^+^ effluxes of PM H^+^-ATPase, indicating that the H^+^ efflux is regulated by the proton pump, and thus, the acidification of the cell wall is important for root elongation (Staal et al., 2011). Similarly, optimal primary root growth and root hair development also require PM H^+^-ATPase to finely regulate H^+^ secretion of root tips (Haruta and Sussman, 2012). In *Arabidopsis*, a total of 11 PM H^+^-ATPase genes are identified and defined as AHA1–AHA11 (*Arabidopsis* PM H^+^-ATPase isoforms) (Palmgren, 2001). Among these isoforms, AHA2 is a housekeeping gene expressed at high levels and is the predominant proton pump in plant roots, contributing to the pH homeostasis and root growth and development (Młodziñska et al., 2014).

Arbuscular mycorrhizal fungi (AMF) form symbiotic association with roots of most terrestrial plants. Host plants transfer carbohydrates to AMF for mycelial growth and spore development; on the other hand, mycorrhizal hyphae enable to absorb nutrients from the soil to the host, thus promoting plant growth and nutrient acquisition (Aggarwal et al., 2011). It has been reported that AMF increased water absorption, nutrient absorption, photosynthesis, root structure, antioxidant defense systems, polyamine and fatty acid homeostasis, osmotic adjustment, aquaporin expression, soil structure, and hormone balance to resist soil water deficit (Wu et al., 2013, 2019; Fernández-Lizarazo and Moreno-Fonseca, 2016; He et al., 2020; Zou et al., 2020). These reactions are the result of a combination of nutritional, physical, and cellular effects. In addition, studies have shown that hyphal H^+^ effluxes and spores growth rate were stimulated during the presymbiotic development of *Gigaspora margarita*, which is related to phosphorus and sucrose deficiency (Ramos et al., 2008b). And, in the symbiosis of maize and mycorrhizal fungi, the H^+^ pump activity of the host plant was differently regulated by AMF (Ramos et al., 2009). Liu et al. (2020) reported that the tomato AM-specific H^+^-ATPase, *SIHA8*, was evolutionarily conserved in maintaining arbuscule development. In tobacco, Gianinazzi-Pearson et al. (2000) reported the expression of *H^+^-ATPase* located in cortical parenchyma of AMF-colonized cells only. In addition, two P-type HA genes *GmHA5* and *GmPMA1* were encoded in *Glomus mosseae* (Ferrol et al., 2000; Requena et al., 2003). However, the relationship between AMF and *PM H^+^-ATPase* gene (especially *AHA2*) expressions of host plants under water deficit is not yet known.

Here, we hypothesized that AMF potentially regulated PM H^+^-ATPase activity and *AHA2* gene expression under DS, thus promoting root growth and nutrient (NH_4_^+^) acquisition to tolerate soil drought. To confirm the above hypothesis, trifoliate orange (*Poncirus trifoliata* L. Raf., a drought-sensitive citrus rootstock) was selected and inoculated with an AMF *Funneliformis mosseae*. Leaf gas exchange, root morphology, H^+^-ATPase activity and *AHA2* gene expression in leaf and root, soil pH value, and ammonium content of trifoliate orange were determined under well-watered (WW) and DS.

## Materials and Methods

### Experimental Design

The experiment was a completely random design with two soil water regimes (WW and DS) and two AMF inoculations (+AMF and -AMF). Each treatment had eight replicates, in a total of 32 pots (three seedlings per pot).

### Mycorrhizal Fungal Inoculants

*Funneliformis mosseae* (Nicol. and Gerd.) C. Walker & A. Schüβler [BGC XJ02] was selected. The arbuscular mycorrhizal fungal strain was provided by the Bank of Glomales in China (BGC) and propagated with white clover as its host for 3 months under greenhouse and potted conditions. At harvest time, the shoot of the white clover was removed, and roots and growth substrates were collected as the mycorrhizal inoculants, which contained the fungal spores (22 spores/g), sporocarps, mycorrhizal hyphae, and colonized roots.

### Plant Culture

The four-leaf-old trifoliate orange seedling that was grown in autoclaved sand in an incubator at 26°C was transferred into a 1.4-L plastic pot containing 1.2 kg of autoclaved soil and sand (5:3, vol/vol). The soil properties had been described in detail in Cheng et al. (2021). At the time of transplanting the seedlings, 100 g of mycorrhizal inoculums was applied into trifoliate orange seedlings as the AMF treatment. The non-AMF treatment received 100 g autoclaved (0.11 MPa, 121°C, 1.5 h) mycorrhizal inoculums plus 2-mL filtrates (25-μm filters) of inoculums.

Before soil drought began, potted soil water maintained 75% maximum field water-holding capacity (corresponding to WW) by weighing the pots every day. After 11 weeks, half of the mycorrhizal and non-mycorrhizal plants were exposed to the 55% maximum field water-holding capacity (corresponding to DS) for 8 weeks, and the other half was continued to grow in soil with WW status for 8 weeks. Thereafter, these treated seedlings were harvested. During the experiment, positions of the pots were swapped weekly to reduce the environmental impact. The plants were grown in a greenhouse from March 24 to August 5, 2019, where the day/night temperature was 28°C/21°C, relative humidity 68%, and photon flux density 880 μmol/m^2^ per second.

### Determination of Leaf Gas Exchange

On a sunny morning from 9:00 to 11:00 am before the plants were harvested, leaf gas exchange was determined using a Li-6400 Portable Photosynthesis System (Li-Cor Inc., Lincoln, NE, United States) in the fully extended leaf. Photosynthetic rate (Pn), stomatal conductance (g_*s*_), intercellular CO_2_ concentration (Ci), transpiration rate (Tr), and leaf temperature were recorded after steady state of gas exchange.

### Determination of Root Mycorrhizal Colonization and Root Morphology

Root mycorrhizas were stained by 0.05% trypan blue as described by Phillips and Hayman (1970). Root mycorrhizal colonization degree was assessed as the percentage of root lengths colonized by mycorrhizal fungi against total observed root lengths. The intact roots were carefully scanned by the Epson Perfection V700 Photo Dual Lens System (J221A, Seiko Epson Corporation, Jakarta, Indonesia), and then the obtained root figures were analyzed with a WinRHIZO professional software (Regent Instruments Inc., Quebec, Canada) for root area, volume, and average diameter.

### Determination of H^+^-ATPase Activity in Leaf and Root

The activity of H^+^-ATPase in leaf and root was determined by the enzyme-linked immunosorbent assay (ELISA). A 0.3-g fresh plant sample was ground under liquid nitrogen, incubated with phosphate buffer (pH 7.4), and centrifuged at 8,000*g* for 30 min. The supernatant was collected, and the H^+^-ATPase activity was assayed by the H^+^-ATPase ELISA kits (ml73629, Shanghai Enzyme-linked Biotechnology Co., Ltd., Shanghai, China) according to the user’s guide.

### Polymerase Chain Reaction Amplification and Sequence Analysis of PtAHA2 (a PM H^+^-ATPase Gene)

Based on the known *AHA2* (*Arabidopsis* PM H^+^-ATPase isoform 2) gene sequence (AT4G30190) in *Arabidopsis thaliana* and the BLAST result of sweet orange genome^1^, a pair of primers (F: 5′-CCCAACAAGCTAGAA GAGAAAAAG-3′; R: 5′-CGCGAGTAATGTTTTTTCCTTCTG-3′) were designed with the most homologous gene sequence (Cs4g03700.1) for open reading frame amplification.

Total RNA was extracted and purified from leaves and roots of trifoliate orange seedlings with the EASY spin Plus plant RNA kit (RN 38, Aidlab Biotecnolohies Co., Ltd., Beijing, China). After determining the concentration and purity of the isolated RNA by spectrophotometers at 260 and 280 nm, the RNA was reverse-transcribed into cDNA using the kit PrimeScript^TM^ RT reagent kit with a gDNA eraser (PK02006, TaKaRa Bio. Inc., Tokyo, Japan). The polymerase chain reaction (PCR) amplification was initiated using the Planta max super-fidelity DNA polymerase kit (Vazyme Biotech Co., Ltd., Nanjing, China) and recovered the destination fragment by the universal DNA purification kit (TianGen Biotech Beijing Co., Ltd., Beijing, China). The pEASY^®^-Blunt Zero Cloning Kit (Beijing TransGene Biotech Co., Ltd., Beijing, China) was used for the ligation and transformation of the target fragment. Afterward, it was screened on LB plates coated with kanamycin, and positive clones were isolated by PCR for sequencing. DNAMAN5.2.2 (Lynnon Biosoft, Quebec, Canada) and MEGA 7^2^ were used to analyze the sequence alignment and the phylogenetic tree analysis, respectively.

### Relative Expressions of *PtAHA2*

The primer was designed based on the predicted PM PtAHA gene sequence. Accumulation of transcript was measured by quantitative real-time PCR (Light Cycler480 System, Roche Diagnostics, Switzerland) using the TB Green premix Ex^TM^ TaqII for fluorescence quantitative analysis. Gene-specific primer sequences of *PtAHA2* gene were as follows: F: 5′-GATGGTAAATGGAGTGAGGAAGAAG-3′; R: 5′-GGATTTTTGGTGACAGGAAGAGAT T-3′. The β-actin gene (Cs1g05000; forward: 5′-CCGACCGTATGAGCAAGGAAA-3′; reverse: 5′-TTCCTGTGGACAATGGATGGA-3′) was used as the housekeeping gene. The expression level of *PtAHA2* was normalized to the expression level of non-AMF plants exposed to WW. The 2^–ΔΔ*CT*^ method was used to calculate the relative quantification (Livak and Schmittgen, 2001).

### Determination of Soil pH Value

A 10-g air-dried soil sample through 2-mm sieves was mixed with 25 mL distilled water without CO_2_ (soil:water = 1:2.5) and shaken well with a magnetic stirrer. After 30 min, the pH value of the supernatant was determined using a pH meter (pH828+, Smart Sensor, Dongguan, China) after precalibration.

### Determination of Ammonium Content in Leaf and Root

Ammonium content in leaf and root was determined by the ninhydrin method described by Tang (1999). A 0.5-g fresh sample was ground with 5 mL of 10% acetic acid and filtered. Afterward, 2-mL filtrate was mixed with 3 mL of the ninhydrin reagent (C_9_H_4_O_3_⋅H_2_O)and 0.1 mL of 1% ascorbic acid solution at 100°C for 15 min. After cooling, the absorbance of the solution was determined at 580 nm.

### Statistical Analysis

All the data were analyzed with the analysis of variation according to SAS software (SAS Institute, Inc., Cary, NC, United States), and significant differences between treatments were compared by the Duncan multiple-range tests at *p* = 0.05 level. The Pearson correlation coefficient between two specified variables was analyzed with SAS software.

## Results

### Changes in Root Mycorrhizal Colonization and Root Morphology

**FIGURE 1:**
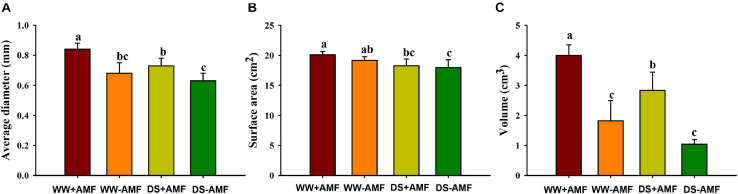
Effects of *Funneliformis mosseae* on root average diameter **(A)**, root surface area **(B)**, and root volume **(C)** of trifoliate orange (*Poncirus trifoliata*) seedlings subjected to well-watered (WW) and drought stress (DS). Data (means ± SD, *n* = 4) followed by different letters above the bars indicate significant differences (*P* < 0.05) between treatments.

Mycorrhizal fungal colonization was not found in the roots of uninoculated plants, whereas the root mycorrhizal colonization rate of the inoculated plants was 46.91% ± 1.86% under WW and 34.11% ± 7.10% under DS, respectively. The DS treatment significantly reduced root mycorrhizal colonization, compared with the WW treatment. Drought treatment also inhibited root morphology to a certain extent, and *F. mosseae* inoculation partly mitigated the inhibitive effect (Figures 1A–C). Compared with non-AMF treatment, AMF inoculation increased root average diameter and volume, respectively, by 23.53 and 119.78% under WW and by 15.87 and 172.11% under DS, respectively (Figures 1A,C). Mycorrhizal fungal treatment did not significantly affect root surface area, regardless of soil water regimes (Figure 1B).

### Changes in Leaf Gas Exchange

Drought treatment significantly decreased leaf Pn, g_*s*_, Ci, and Tr, compared with WW treatment (Table 1). Compared with non-mycorrhizal fungal plants, *F. mosseae*–inoculated plants recorded significantly higher leaf Pn, g_*s*_, Ci, and Tr by 118.80, 237.93, 45.61, and 237.70% under WW and by 141.15, 103.49, 60.70, and 134.04% under DS, respectively. In addition, leaf temperature was markedly reduced by *F. mosseae* inoculation by 1.67% under WW only.

**TABLE 1:** Effects of *Funneliformis mosseae* on leaf gas exchange of trifoliate orange (*Poncirus trifoliata*) seedlings subjected to well-watered (WW) and drought stress (DS).

Treatments	Pn (μmol/m^2^ per second)	g_*s*_ (μmol/m^2^ per second)	Ci (μmol/mol)	Tr (mmol/m^2^ per second)	Leaf temperature (°C)
WW + AMF	5.47 ± 0.90a	96.14 ± 5.80a	339.58 ± 18.28a	2.06 ± 0.47a	34.01 ± 0.62b
WW-AMF	2.50 ± 0.39c	28.45 ± 4.60c	233.22 ± 18.03c	0.61 ± 0.26c	34.69 ± 0.89a
DS + AMF	4.63 ± 1.05b	47.76 ± 6.84b	301.96 ± 10.30b	1.10 ± 0.42b	34.57 ± 0.62a
DS-AMF	1.92 ± 0.68d	23.47 ± 4.41d	187.90 ± 16.53d	0.47 ± 0.10c	34.64 ± 0.82a

*Data (means ± SD, n = 4) followed by different letters in the column indicate significant differences (P < 0.05) between treatments.*

### Changes in Leaf and Root H^+^-ATPase Activity

**FIGURE 2:**
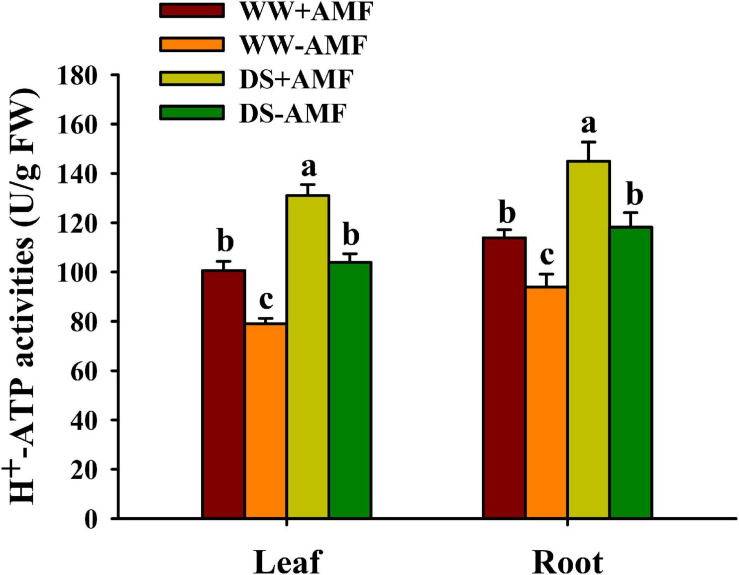
Effects of *Funneliformis mosseae* on leaf and root H^+^-ATP activities of trifoliate orange (*Poncirus trifoliata*) seedlings subjected to well-watered (WW) and drought stress (DS). Data (means ± SD, *n* = 4) followed by different letters above the bars indicate significant differences (*P* < 0.05) between treatments.

Soil drought treatment significantly increased H^+^-ATPase activity in leaf and root, irrespective of AMF inoculation or not (Figure 2). On the other hand, AMF colonization distinctly enhanced H^+^-ATPase activity in leaf by 27.37 and 26.06% and in root by 22.14 and 22.61% under WW and DS conditions, respectively, compared with non-AMF colonization.

### Sequence Analyses of PtAHA2

**FIGURE 3:**
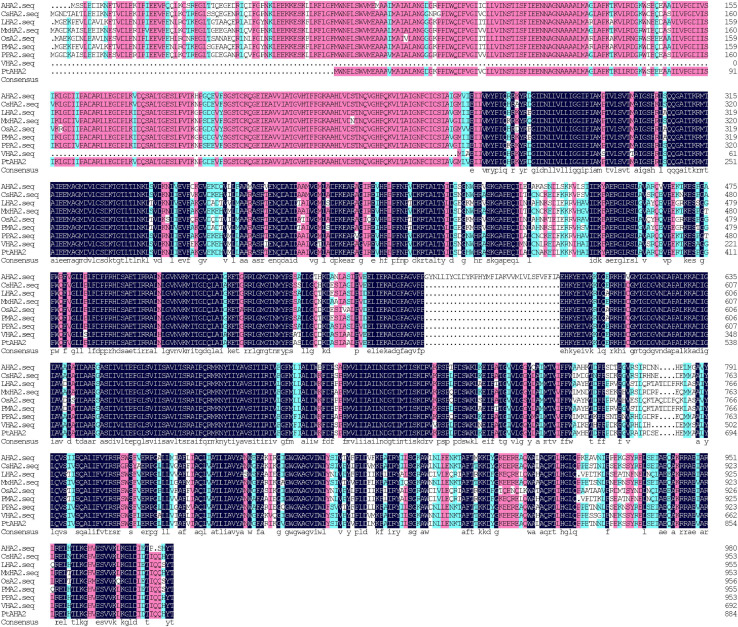
Sequence alignments of *PtAHA2* with *HA2* proteins from other plants. *AHA2*: *Arabidopsis thaliana*; *CsHA2*: *Cucumis sativus*; *LHA2*: *Lycopersicon esculentum*; *MxHA2*: *Malus xiaojinensis*; *OSA2*: *Oryza sativa*; *PPA2*: *Prunus persica*; *VHA2*: *Vicia faba*; *PtAHA2*: *Poncirus trifoliata*.

A total of 2,965 bp in PM H^+^-ATPase gene (*PtAHA2*) encoding 885 amino acids were cloned from trifoliate orange, based on sweet orange database and homologous cloning. A GenBank accession number of *PtAHA2* was MW239123. The sequencing of the *PtAHA2* showed that the protein sequence homology between trifoliate orange and sweet orange was 99.66% by DNAMAN (Supplementary Material 1), suggesting that trifoliate orange has a high homology with sweet orange. In addition, multiple sequence alignment indicated that the sequence homology of *PtAHA2* proteins with other *AHA2* families was 83.72% (Figure 3), indicating that HA2-type proteins have high sequence conservation. Based on the phylogenetic tree analysis of *PtAHA2*, five H^+^-ATPase genes in *Arabidopsis* (*AtAHA2*, *AtAHA6*, *AtAHA7*, *AtAHA4*, and *AtAHA10*) and five H^+^-ATPase genes in *Oryza sativa* (*OsAHA7*, *OsAHA6*, *OsAHA8*, *OsAHA1*, and *OsAHA9*) from families I to V by MEGA 7, *PtAHA2* belonged to subfamily I, which was clustered with *OsAHA7* and *AtAHA2* (Supplementary Material 2).

### Changes in *PtAHA2* Gene Expression

**FIGURE 4:**
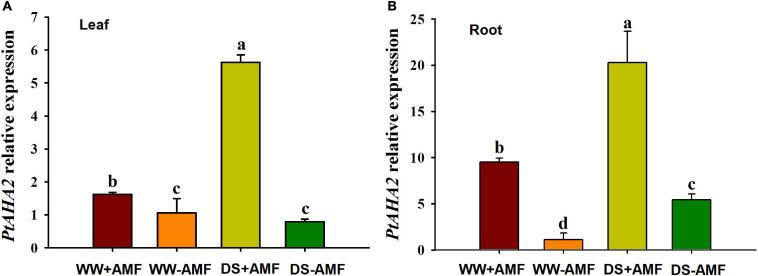
Effects of *Funneliformis mosseae* on relative expression of leaf **(A)** and root **(B)** plasma membrane H^+^-ATPase gene *PtAHA2* in trifoliate orange (*Poncirus trifoliata*) seedlings subjected to well-watered (WW) and drought stress (DS). Data (means ± SD, *n* = 3) followed by different letters above the bars indicate significant differences (*P* < 0.05) between treatments.

Compared with WW treatment, DS treatment did not affect the *PtAHA2* expression in leaves of non-mycorrhizal plants, while it induced the expression of *PtAHA2* in leaves of mycorrhizal seedlings and in roots of mycorrhizal and non-mycorrhizal seedlings (Figure 4). Moreover, the expression of *PtAHA2* in roots was relatively higher than in leaves. In addition, leaf and root *PtAHA2* gene expression was increased under mycorrhization by 1.62- and 9.50-fold under WW and by 5.62- and 20.92-fold under DS, respectively.

### Changes in Soil pH Value

**FIGURE 5:**
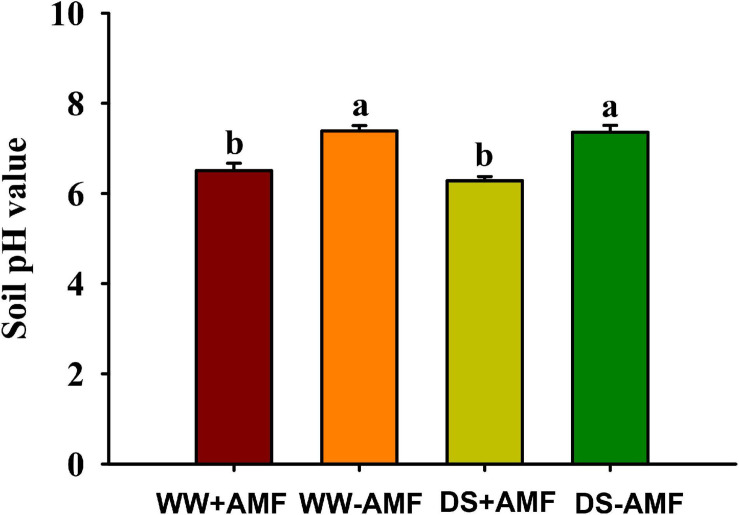
Effects of *Funneliformis mosseae* on soil pH value in trifoliate orange (*Poncirus trifoliata*) seedlings subjected to well-watered (WW) and drought stress (DS). Data (means ± SD, *n* = 4) followed by different letters above the bars indicate significant differences (*P* < 0.05) between treatment.

Soil pH value was affected by mycorrhization, but not soil drought treatment (Figure 5). Compared with non-AMF inoculation, AMF inoculation significantly reduced soil pH value by 12.04 and 14.67% under WW and DS, respectively.

### Changes in Leaf and Root Ammonium Content

**FIGURE 6:**
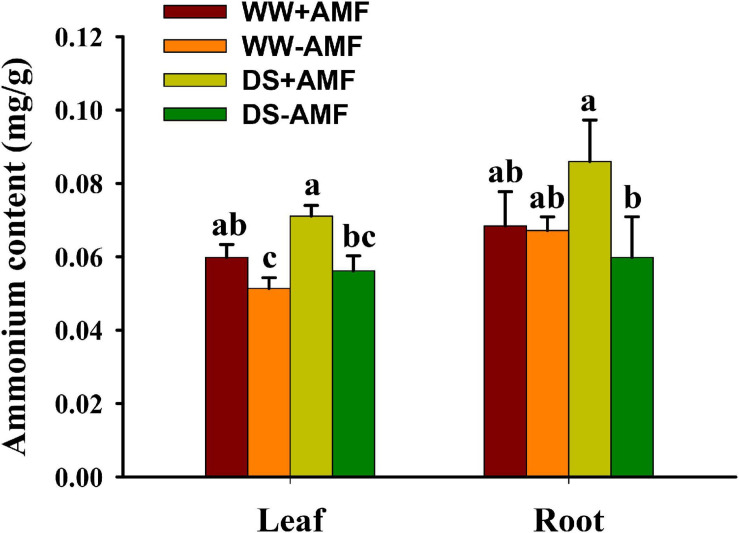
Effects of *Funneliformis mosseae* on leaf and root ammonium contents in trifoliate orange (*Poncirus trifoliata*) seedlings subjected to well-watered (WW) and drought stress (DS). Data (means ± SD, *n* = 4) followed by different letters above the bars indicate significant differences (*P* < 0.05) between treatment.

Leaf and root ammonium contents were not significantly affected by soil DS treatment, irrespective of mycorrhizal and non-mycorrhizal plants (Figure 6). AMF colonization significantly enhanced the absorption of ammonium in leaf and root by 26.79 and 43.33% under DS, respectively, compared with non-AMF colonization. Under WW, AMF-inoculated plants recorded 17.65% significantly higher leaf ammonium content than non-AMF-inoculated plants, although no significant difference in root ammonium content was found between AMF- and non-AMF-inoculated plants.

### Correlation Studies

**FIGURE 7:**
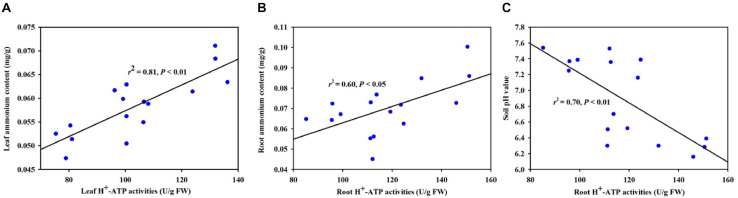
Linear regression between H^+^-ATPase activity and ammonium content in leaf **(A)** and root **(B)** and between root H^+^-ATPase activity and soil pH value **(C)** (*n* = 16).

Based on the correlation analysis between H^+^-ATPase and selective variables, we found that leaf H^+^-ATPase activity was significantly (*P* < 0.01) positively correlated with leaf ammonium content (Figure 7A). Root H^+^-ATPase activity was significantly (*P* < 0.05) positively correlated with root ammonium content (Figure 7B) and negatively (*P* < 0.01) correlated with soil pH value (Figure 7C).

## Discussion

Earlier studies showed that mycorrhizal plants had greater leaf gas exchange capacity than non-mycorrhizal plants subjected to abiotic stress (e.g., DS and salt stress) (Porcel and Ruiz-Lozano, 2004; Xiong et al., 2007; Augé et al., 2015). Our study also confirmed that Pn, g_*s*_, Ci, and Tr of mycorrhizal trifoliate orange seedlings were significantly higher than that of non-mycorrhizal seedlings under both WW and DS, implying that mycorrhizal symbiosis has the ability to improve leaf gas exchange of the host under DS. As a result, mycorrhizal plants might produce more carbohydrates for the growth of both the host and the mycorrhizal fungus and also stimulate the transport of water exposed to DS (Hadian-Deljou et al., 2020). In addition, *H^+^-ATPase* genes such as *AHA2* were highly expressed in guard cells of *Arabidopsis* for stomatal opening (Ueno et al., 2005). Overexpression of *AHA2* in guard cells of *Arabidopsis* could manipulate stomatal opening, thereby improving plant growth (Wang et al., 2014). In the present study, we concluded that the up-regulated expression of *PtAHA2* in leaf by mycorrhization may be associated with better leaf gas exchange of mycorrhizal plants, especially stomatal opening under DS.

Plant roots contact the soil directly to respond to environmental stress (Ingrain and Malamy, 2010; Franco et al., 2011). The present results showed that the inoculation of *F*. *mosseae* substantially improved root average diameter and volume under both WW and DS, compared with the non-AMF inoculation. Analogous results have been reported by Zou et al. (2017) in trifoliate orange. It is documented that PM AHA2 is very important for root growth and development, as shown by shorter and lower primary and lateral root length in *aha2* mutant lines of *A. thaliana* versus wild-type plants (Młodziñska et al., 2014). Hence, under WW and DS, *F*. *mosseae* inoculation induced the activity and gene expression of *PtAHA2* in roots, which potentially stimulates the growth of primary and lateral root, resulting in greater root volume in mycorrhizal plants. As shown in this study, the rhizosphere of mycorrhizal trifoliate orange plants had a low pH environment (Figure 7), and indoleacetic acid can be protonized to expand the membrane for transport into the cells, thereby accelerating root growth (Petrasek and Friml, 2009). Therefore, mycorrhizal plants have strong adaptability by optimizing the root morphology to potentially mitigate drought damage (Chu et al., 2014).

In this study, we observed that soil drought treatment dramatically increased leaf and root H^+^-ATPase activity in AM and non-AM plants, indicating that trifoliate orange seedlings could rapidly adapt to soil drought environment by enhancing the activity of H^+^-ATPase in leaf and root. Furthermore, mycorrhizal trifoliate orange seedlings recorded significantly higher H^+^-ATPase activity in leaf and root under both WW and DS conditions, suggesting that H^+^-ATPase activity of trifoliate orange could be induced by AMF.

The PM H^+^-ATPase is a large gene family that exhibits expression overlap and functional redundancy, but *AHA1* and *AHA2* are highly expressed in almost all tissues and organs (Haruta et al., 2010). Studies by Haruta et al. (2010) showed that single knockout of *AHA1* or *AHA2* did not represent an obvious phenotype, but double knockout is lethal. In our study, *PtAHA2* expression was higher in roots than in leaves, possibly because AHA2 had a strong signal in epidermal and cortex cells of roots, as well as in phloem, xylem, and root hairs (Gianinazzi-Pearson et al., 2000; Fuglsang et al., 2007). The *PtAHA2* expression was induced by soil drought, especially in roots. The AMF-inoculated seedlings recorded dramatically higher expression of *PtAHA2* gene in leaf and root than the non-AMF-inoculated seedlings. And the induced expression of *PtAHA2* under mycorrhization was more prominent under DS than under WW. Thus, *PtAHA2* showed an AMF-specific expression profile. Liu et al. (2016) also analyzed the relative transcript of levels of four tomato *H^+^-ATPase* (HA) genes (*SlHA1*, *SlHA2*, *SlHA4*, and *SlHA8*) and found that *SlHA1* and *SlHA4* in leaf and root were not affected by mycorrhization, *SlHA2* was up-regulated by mycorrhization only in root, and *SlHA8* was expressed in root and activated by mycorrhization. This implied that plant *HA* expression could be regulated by mycorrhization, dependent on host plants and HA homologous genes. Further work should analyze the expression of *HA* genes in both host plants and mycorrhizal fungi (e.g., *GmHA5* and *GmPMA1*) in response to DS, which is cooperative or competitive.

In our study, we found lower soil pH value in rhizosphere of mycorrhizal plants versus non-mycorrhizal plant, regardless of soil water regimes. Additionally, soil pH value was significantly negatively correlated with root H^+^-ATPase activity, because H^+^-ATPase releases protons into rhizosphere, resulting in a low pH environment (Chen et al., 2017). Acidic rhizosphere under environmental stress conditions is important to the nutrient availability in soil (Ramos et al., 2008a). The H^+^ electrochemical gradient generated by H^+^-ATPase provides driving forces for nutrients and solutes on the symbiotic membranes and participates in nutrient transfer (Garry et al., 2007). Liu et al. (2020) further analyzed the *SlHA8* expression in tomato and found that the *SlHA8* expression was essential for arbuscule development and symbiotic N uptake. In tobacco, *H^+^-ATPase* gene was expressed in AMF-colonized cortical cells only (Gianinazzi-Pearson et al., 2000). In *Medicago truncatula*, a *ha1-2* mutant was isolated and impaired in arbuscule development, but not in root colonization of *Rhizophagus irregularis* (Krajinski et al., 2014). The AMF-induced HA gene was mainly localized in arbuscule-containing cells, where it is the nutrient unloading interface between AMF and hosts. When H^+^ ions are released into rhizosphere, plants also absorb ammonium ions into the root to maintain the charge balance (Motte and Beeckman, 2020). Our study also showed that the inoculation with *F*. *mosseae* dramatically improved ammonium content in leaf under WW and DS and in root under DS, as compared with non-AMF inoculation. Moreover, H^+^-ATPase activity was significantly positively correlated with ammonium content. Sun et al. (1999) observed that the activation of the proton pump in mycorrhizal hyphae under drought conditions regulated the rhizospheric microenvironment, resulting in inorganic ions accumulation in the soil. Therefore, the increase of ammonium ions in mycorrhizal plants is related to the induced expression of host *H^+^-ATP* gene by mycorrhizal fungi, in addition to mycorrhizal hyphal absorption (Johansen et al., 2010).

## Conclusion

In short, we cloned a H^+^-ATPase gene of trifoliate orange, *PtAHA2*, which is evolutionarily conserved. The *PtAHA2* expression distinctly increased under mycorrhization and soil DS, along with higher H^+^-ATPase activity in AM plants versus non-AM plants. The increase in *PtAHA2* gene expression and H^+^-ATPase activity under mycorrhization was closely associated with root growth (e.g., root volume and average diameter), nutrient acquisition (ammonium), and leaf gas exchange (e.g., stomatal conductance), which are critical for enhanced drought tolerance of plants. Further analysis of mycorrhiza-specific *PtAHA2* on mycorrhizal plants by molecular techniques such as gene knockout and screening for upstream transcription factors can help us understand the function of *PtAHA2* in response to mycorrhization and DS.

## Data Availability Statement

The original contributions presented in the study are included in the article/Supplementary Material, further inquiries can be directed to the corresponding authors.

## Author Contributions

H-QC and Q-SW conceived and designed the experiments. H-QC performed the experiments. H-QC, Y-NZ, Q-SW, and KK analyzed the data. H-QC and Y-NZ prepared the figures. H-QC wrote the manuscript. Q-SW and KK revised the manuscript. All authors contributed to the article and approved the submitted version.

## Conflict of Interest

The authors declare that the research was conducted in the absence of any commercial or financial relationships that could be construed as a potential conflict of interest.

## Footnotes

1 http://citrus.hzau.edu.cn/orange/index.php

2 www.megasoftware.net
