# Supplementary material for: Arbuscular Mycorrhizal Fungi Alleviate Drought Stress in Trifoliate Orange by Regulating H+-ATPase Activity and Gene Expression
Source: Front Plant Sci. 2021 Mar 25;12:659694. doi: 10.3389/fpls.2021.659694 (PMC8027329; doi:10.3389/fpls.2021.659694)
Supplement: Supplementary file 1 [file Data_Sheet_1.PDF]

```
PtAHA2      MWNPLSWVMEAAAVMAIALANGDGKPPDWQDFVGIVCLLVINSTISFIEENNAGNAAAALMAGLAPKTKLLRDGKWSEEEAAILVPGDIISIKLGDIIPADARLLEGDPLKVDQSALTGESLPVTKNPGDEVFSGSTCKQGEIEAVVIATGVHTFFGKAA   160
4g03700.1   MWNPLSWVMEAAAVMAIALANGEGKPPDWQDFVGIVCLLVINSTISFIEENNAGNAAAALMAGLAPKTKLLRDGKWSEEEAAILVPGDIISIKLGDIIPADARLLEGDPLKVDQSALTGESLPVTKNPGDEVFSGSTCKQGEIEAVVIATGVHTFFGKAA   160
Consensus   mwnplswvmeaaavmaialang gkppdwqdfvgivcllvinstisfieennagnaaaalmaglapktkllrdgkwseeeaailvpgdiisiklgdiipadarllegdplkvdqsaltgeslpvtknpgdevfsgstckqgeieavviatgvhtffgkaa

PtAHA2      HLVDSTNQVGHFQKVLTAIGNFCICSIAVGMLVEIIVMYPIQHRKYRDGIDNLLVLLIGGIPIAMPTVLSVTMAIGSHRLSQQGAITKRMTAIEEMAGMDVLCSDKTGTLTLNKLSVDKNLIEVFAKGVEKDHVMLLAARASRTENQDAIDAAIVGMLAD   320
4g03700.1   HLVDSTNQVGHFQKVLTAIGNFCICSIAVGMLVEIIVMYPIQHRKYRDGIDNLLVLLIGGIPIAMPTVLSVTMAIGSHRLSQQGAITKRMTAIEEMAGMDVLCSDKTGTLTLNKLSVDKNLIEVFAKGVEKDHVMLLAARASRTENQDAIDAAIVGMLAD   320
Consensus   hlvdstnqvghfqkvltaignfcicsiavgmlveiivmypiqhrkyrdgidnllvlliggipiamptvlsvtmaigshrlsqqgaitkrmtaieemagmdvlcsdktgtltlnklsvdknlievfakgvekdhvmllaarasrtenqdaidaaivgmlad

PtAHA2      PKEARAGIREVHFFPFNPVDKRTALTYIDSDGHWHRASKGAPEQILALCNAKEDLKKKVHAIIDKYAERGLRSLAVARQEVPERTKESPGGPWQFVGLLPLFDPPRHDSAETIRRALNLGVNVKMITGDQLAIAKETGRRLGMGTNMYPSASLLGQDKDA   480
4g03700.1   PKEARAGIREVHFFPFNPVDKRTALTYIDSDGHWHRASKGAPEQILALCNAKEDLKKKVHAIIDKYAERGLRSLAVARQEVPERTKESPGGPWQFVGLLPLFDPPRHDSAETIRRALNLGVNVKMITGDQLAIAKETGRRLGMGTNMYPSASLLGQDKDA   480
Consensus   pkearagirevhffpfnpvdkrtaltyidsdghwhraskgapeqilalcnakedlkkkvhaiidkyaerglrslavarqevpertkespggpwqfvgllplfdpprhdsaetirralnlgvnvkmitgdqlaiaketgrrlgmgtnmypsasllgqdkda

PtAHA2      SIAALPVEELIEKADGFAGVFPEHKYEIVKKLQERKHICGMTGDGVNDAPALKKADIGIAVADATDAARGASDIVLTEPGLSVIISAVLTSRAIFQRMKNYTIYAVSITIRIVFGFMFIALIWKFDFSPFMVLIIAILNDGTIMTISKDRVKPSPLPDSW   640
4g03700.1   SIAALPVEELIEKADGFAGVFPEHKYEIVKKLQERKHICGMTGDGVNDAPALKKADIGIAVADATDAARGASDIVLTEPGLSVIISAVLTSRAIFQRMKNYTIYAVSITIRIVFGFMFIALIWKFDFSPFMVLIIAILNDGTIMTISKDRVKPSPLPDSW   640
Consensus   siaalpveeliekadgfagvfpehkyeivkklqerkhicgmtgdgvndapalkkadigiavadatdaargasdivltepglsviisavltsraifqrmknytiyavsitirivfgfmfialiwkfdfspfmvliiailndgtimtiskdrvkpsplpdsw

PtAHA2      KLKEIFATGVVLGGYLALMTVIFFWAMHETDFFPDKFGVRAIRDSEHEMMAALYLQVSIVSQALIFVTRSRSWSFLERPGLLLVTAFVIAQLVATLIAVYANWGFARIKGVGWGWAGVIWLYSIVFYVPLDLMKFAIRYILSGKAWLNLLENKTAFTTKK   800
4g03700.1   KLKEIFATGVVLGGYLALMTVIFFWAMHETDFFPDKFGVRAIRDSEHEMMAALYLQVSIVSQALIFVTRSRSWSYLERPGLLLVTAFVLAQLVATLIAVYANWGFARIKGVGWGWAGVIWLYSIVFYVPLDLMKFAIRYILSGKAWLNLLENKTAFTTKK   800
Consensus   klkeifatgvvlggylalmtviffwamhetdffpdkfgvrairdsehemmaalylqvsivsqalifvtrsrsws lerpglllvtafv aqlvatliavyanwgfarikgvgwgwagviwlysivfyvpldlmkfairyilsgkawlnllenktafttkk

PtAHA2      DYGKEEREAQWALAQRTLHGLQPPETNNLFPEKSSYRELSEIAEQAKRRAEVARLRELHTLKGHVESVVKLKGLDIDTIQQHYT   884
4g03700.1   DYGKEEREAQWALAQRTLHGLQPPETNNLFPEKSSYRELSEIAEQAKRRAEVARLRELHTLKGHVESVVKLKGLDIDTIQQHYT   884
Consensus   dygkeereaqwalaqrtlhglqppetnnlfpekssyrelseiaeqakrraevarlrelhtlkghvesvvklkgldidtiqqhyt
```

Supplementary material 1. Alignment of the protein of a plasma membrane H^{+}ATPase gene (*PtAHA2*) in trifoliate orange and sweet orange (4g03700.1)

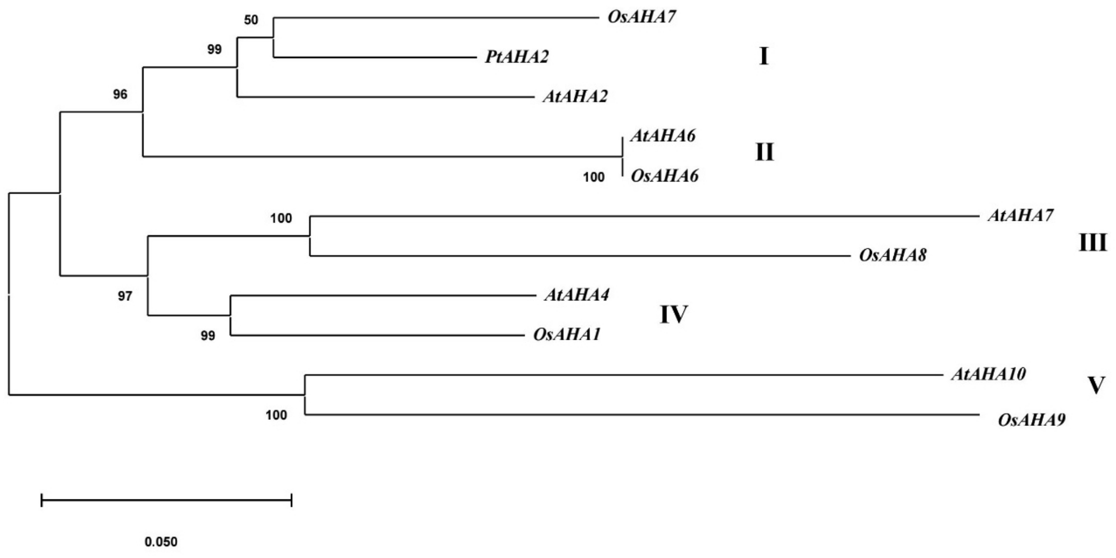

Supplementary material 2. Evolutionary tree analysis which gene family of PtAHA2 belongs to from I-V. *Arabidopsis thaliana* (*AtAHA2*, *AtAHA6*, *AtAHA7*, *AtAHA4* and *AtAha10)* and *Oryza sativa* (*OsAHA7*, *OsAHA6, OsAHA8*, *OsAHA1* and *OsAHA9*).
